# IP3 accumulation and/or inositol depletion: two downstream lithium's effects that may mediate its behavioral and cellular changes

**DOI:** 10.1038/tp.2016.217

**Published:** 2016-12-06

**Authors:** Y Sade, L Toker, N Z Kara, H Einat, S Rapoport, D Moechars, G T Berry, Y Bersudsky, G Agam

**Affiliations:** 1 Department of Clinical Biochemistry and Pharmacology, Ben-Gurion University of the Negev, Beer-Sheva, Israel; 2 Psychiatry Research Unit, Ben-Gurion University of the Negev, Beer-Sheva, Israel; 3 Faculty of Health Sciences, Ben-Gurion University of the Negev, Beer-Sheva, Israel; 4 Mental Health Center, Beer-Sheva, Israel; 5 Department of Psychiatry and Centre for High-Throughput Biology, University of British Columbia Vancouver, BC, Canada; 6 School of Behavioral Sciences, Tel Aviv-Yaffo Academic College, Tel Aviv, Israel; 7 Brain Physiology and Metabolism Section, Laboratory of Neurosciences, National Institute on Aging, National Institutes of Health, Bethesda, MD, USA; 8 Johnson & Johnson Pharmaceutical Research and Development, Beerse, Belgium; 9 Metabolism Program Division of Genetics, Children's Hospital Boston, Harvard Medical School, Boston, MA, USA

## Abstract

Lithium is the prototype mood stabilizer but its mechanism is still unresolved. Two hypotheses dominate—the consequences of lithium's inhibition of inositol monophosphatase at therapeutically relevant concentrations (the ‘inositol depletion' hypothesis), and of glycogen-synthase kinase-3. To further elaborate the inositol depletion hypothesis that did not decisively determine whether inositol depletion *per se*, or phosphoinositols accumulation induces the beneficial effects, we utilized knockout mice of either of two inositol metabolism-related genes—*IMPA1* or *SMIT1*, both mimic several lithium's behavioral and biochemical effects. We assessed *in vivo,* under non-agonist-stimulated conditions, ^3^H-inositol incorporation into brain phosphoinositols and phosphoinositides in wild-type, lithium-treated, *IMPA1* and *SMIT1* knockout mice. Lithium treatment increased frontal cortex and hippocampal phosphoinositols labeling by several fold, but decreased phosphoinositides labeling in the frontal cortex of the wild-type mice of the *IMPA1* colony strain by ~50%. Inositol metabolites were differently affected by *IMPA1* and *SMIT1* knockout. Inositoltrisphosphate administered intracerebroventricularly affected bipolar-related behaviors and autophagy markers in a lithium-like manner. Namely, IP_3_ but not IP_1_ reduced the immobility time of wild-type mice in the forced swim test model of antidepressant action by 30%, an effect that was reversed by an antagonist of all three IP_3_ receptors; amphetamine-induced hyperlocomotion of wild-type mice (distance traveled) was 35% reduced by IP_3_ administration; IP_3_ administration increased hippocampal messenger RNA levels of Beclin-1 (required for autophagy execution) and hippocampal and frontal cortex protein levels ratio of Beclin-1/p62 by about threefold (p62 is degraded by autophagy). To conclude, lithium affects the phosphatidylinositol signaling system in two ways: depleting inositol, consequently decreasing phosphoinositides; elevating inositol monophosphate levels followed by phosphoinositols accumulation. Each or both may mediate lithium-induced behavior.

## Introduction

Bipolar disorder (BPD) is a mental illness characterized by severe high and low moods. For ~70 years, lithium salts (lithium, Li) have been the mainstay mood-stabilizing drug. Yet, the drug's therapeutic mechanism at the molecular level has not yet been resolved.^1^ The discovery of the inhibitory effect of therapeutically relevant Li concentration on inositol monophosphatase-1 (IMPase-1)^2^ led to the inositol depletion hypothesis of Li's beneficial effect in BPD.^3^ Needless to say that additional hypotheses have been raised, for example, inhibition of glycogen-synthase-kinase-3 and inhibition of adenylyl-cyclase,^4^ neither of which has been either confirmed or rejected beyond doubt. The inositol depletion hypothesis, dealt with in the present study, suggests that the uncompetitive inhibition of IMPase-1 causes modulation of brain levels of inositol and its metabolites resulting in reduced signaling capacity, but it has not decisively determined whether inositol depletion *per se* or phosphoinositol accumulation induces the drug's beneficial effects. Some studies^5, 6^ suggested that rather than inositol depletion increased brain phosphoinositols levels following IMPase-1 inhibition mediate Li's therapeutic action. Up until now observations related to the inositol depletion hypothesis are inconsistent and do not prove or refute the hypothesis. Observations that support the inositol depletion hypothesis include the following: (i) therapeutically relevant Li concentrations could directly inhibit purified IMPase from different sources;^2^ (ii) Li reduced brain inositol levels^7^ and elevated inositol monophosphate (IP_1_), the substrate of IMPase, in rat cortex;^7, 8^ (iii) Li administration reduced sodium-*myo*-inositol transporter 1 (SMIT1) messenger RNA (mRNA) levels and lowered inositol uptake in astrocyte cultures;^9, 10, 11, 12^ (iv) studies demonstrated Li inhibition of IMPase and elevated IP_1_ levels in rat cortex slices;^13^ (v) some cellular and behavioral effects of Li such as increasing growth cone area,^14^ enhancing autophagy,^15^ suppression of rearing in rats^16, 17^ and hypersensitivity to pilocarpine-induced seizures were reversed by inositol;^16, 17^ (vi) reduced brain inositol levels and increased IP_1_ levels have been reported in BPD patients treated chronically or acutely with Li compared with healthy controls;^18, 19^ (vii) Li treatment of BPD patients reduced phosphatidylinositol bisphosphate (PIP_2_) levels in their platelet membranes;^20^ and (viii) Abbott *et al.*^21^ found that SMIT1 and potassium channel subunits formed complexes and reciprocally regulated each other in the choroid plexus epithelium affecting neuronal excitability. Nevertheless, there are findings that cast some doubt on the inositol depletion hypothesis including: (i) SMIT1 homozygote knockout (KO) mice in which brain inositol levels are ~60% reduced^22^ do not exhibit the expected reduction in brain phosphatidylinositol (PI) level;^23^ (ii) the effect of Li on brain IP_3_ levels is species-specific, resulting in either reduced or increased levels;^24^ (iii) some studies failed to demonstrate reduced brain inositol levels following chronic or acute Li treatment of patients;^25, 26^ (iv) KO mice lacking IMPA1 (encoding for IMPase-1, the brain abundant IMPase that is inhibited by therapeutically relevant Li concentrations^27^) do not exhibit lower frontal cortex or hippocampal inositol levels though brain IMPase activity is decreased by >50%^28, 29^ (v) some behavioral data are also incongruent, for example, acute inositol administration did not block the effect of Li in the mouse forced swim test (FST)^30^ and *myo*-inositol-1-phosphate (MIP) synthase inhibition did not replicate or augment the effects of Li on pilocarpine-induced seizures;^31^ (vi) neither genome-wide association studies nor genomics investigations report findings related either to the PI signaling system or SMIT1 in BPD or Li treatment;^4, 32^ and (vii) genetic studies found variability at IMPA2 but not IMPA1 associated with disease susceptibility, with variation in Li treatment response,^33, 34^ with the emergence of suicide behavior in BPD^35^ and with increased transcription of the IMPA2 allele that harbored a specific haplotype in the frontal cortex of BPD patients.^36^ These inconsistencies may reflect a delicate system homeostasis possibly influenced by experimental conditions, methodologies used and/or animal strains. Whitworth *et al.*^37^ attempted to address these inconsistencies by studying the effect of Li on inositol turnover rather than inositol levels *per se*. They examined the effects of acute and chronic Li treatment in combined extracts of mouse cortex and hippocampus following stimulation with the muscarinic cholinergic agonist, pilocarpine. They reported that acute Li treatment resulted in the accumulation of phosphoinositols that was further enhanced by pilocarpine. Increased phosphoinositides levels were only observed following combined acute treatment with Li and pilocarpine.^37^ Contrarily, when chronic Li treatment was studied, only combined Li+pilocarpine treatment increased phosphoinositols accumulation, whereas phosphoinositides accumulation was observed following Li treatment only.^37^ Others reported that acute Li treatment in rodents resulted in elevated brain phosphoinositols but decreased phosphoinositides levels.^38^ We therefore sought a new approach to assess the inositol depletion hypothesis and to address the above issues.

IMPA1 KO and *Slc5a3* (SMIT1, encoding sodium-*myo*-inositol-transporter) KO mice display behavioral characteristics similar to Li-treated wild-type (WT) mice,^29^ supporting the inositol depletion hypothesis, but somewhat different patterns of brain inositol metabolism.^28, 29, 39^ These mutant mice offer a unique way to further examine the inositol depletion hypothesis. We assessed brain cytosolic ^3^H-phosphoinositols accumulation and incorporation into membrane-bound ^3^H-phosphoinositides following the administration of ^3^H-inositol intracerebroventricularly (ICV) to WT controls, Li-treated WT mice, and IMPA1 and SMIT1 KO mice to address the central question of what are the *in vivo* downstream consequences of Li's inhibition of IMPase-1 (ref. 27) and inositol depletion reduced re-synthesis of phosphoinositides,^3^ accumulation of phosphoinositols^6, 40, 41, 42^ and/or attenuated inositol turnover?^37, 38^ Similar *in vivo* studies in Li-treated mice only were previously reported.^24, 37, 38, 41, 43^

Inositol-monophosphate (IP_1_) accumulation as a result of Li inhibition of IMPase-1 is well established,^3, 37, 38, 40, 41, 44^ but whether, concomitantly, levels of other phosphoinositols and the second messenger IP_3_, in particular, are affected is uncertain. As the first part of the current study demonstrated increased phosphoinositols accumulation in Li-treated and *IMPA1* KO mice, we further studied whether ICV administration of IP_3_ or IP_1_ in liposomes induces Li-like behavior.

IP_3_'s effects are mediated by its receptors (IP_3_Rs—IP_3_R1/2/3).^45^ We found that IP_3_ but not IP_1_ reduced immobility in the FST, an effect that could be reversed by an antagonist of all three IP_3_Rs, xestospongin-C (IP_3_R_ant_). IP_3_ also attenuated amphetamine-induced hyperactivity.

It has been reported that in cells in culture Li upregulated autophagy in an inositol-dependent manner.^15^ Upregulated autophagy had beneficial effects in animal models of affective disorders^46, 47^ and could be mimicked *in vitro* by the administration of IP_3_Rs antagonists or short interfering RNA targeting IP_3_Rs.^48, 49^ *In vivo*, knockdown of IP_3_Rs using specific antisense oligonucleotides led to an antidepressant-like effect in the FST.^50^ Given our result in the first and second part of the study, we tested the effect of IP_3_ on the levels of autophagy markers. IP_3_ induced changes in the autophagy markers Beclin-1 and p62, indicative of enhanced autophagy.

## Materials and Methods

### Blinding

All experiments were carried out by the experimenter (YS aided at times by LT and NK) in a blind manner, namely, blinded to the group an animal/sample belonged to until all results of a given experiment were obtained.

### Animals

SMIT1 and IMPA1 KO mice were generated as described^29^ and as recently detailed.^32^ In short, IMPA1- and SMIT1-KO mice were created on a different C57bl/6 substrain background. Therefore, each of the KOs is maintained in a separate colony. All experiments were approved by the Ben-Gurion University Animal Experimentation Ethics Committee (protocols # IL-02-01-2010, IL-32-05-2012, IL-07-03-2013 and IL-13-04-2013) and were carried out according to the NIH Guide for Care and Use of Laboratory Animals. For experiments not performed in the KO strains, ICR mice (Harlan, Israel or USA) were used. Eight-week-old male mice were used throughout the study. When the KO colonies were used littermate mice were included in all groups of a given experiment.

### Lithium treatment

#### Acute and chronic Li administration

Acute administration: ICR mice (Harlan, USA) or WT mice of the IMPA1 colony were treated with intraperitoneal (i.p.) injection of LiCl at a dose of 3.0 or 10.0 meq/Kg, 10 ml/kg, or a similar volume of saline (control) 24 hours prior to euthanasia and brain extraction for the assessment of phosphoinositols accumulation. Twenty hours prior to death mice were injected ICV with 4 μCi [^3^H]-inositol in 1μl of inositol (20 mg/ml in artificial cerebrospinal fluid (aCSF)) at a rate of 0.5 μl/20 sec. Chronic administration: ICR mice, WT untreated mice, IMPA1 KOs and SMIT1 KOs received powdered rodent chaw (Harlan, Israel). Lithium-treated groups received the same powdered chaw mixed with 0.2% lithium chloride (LiCl) for 5 days followed by 0.4% LiCl for 10 additional days.^51^ All groups received tap water ad libitum and an additional bottle containing 0.9% NaCl to prevent electrolyte imbalance.

### [^3^H]-inositol ICV injection

Mice were anesthetized with 20% isoflurane (diluted in propylene glycol). An incision was made above the bregma and a 25 G needle was used to create a hole in the scalp above the lateral ventricle, 0.2–0.3 mm posterior to bregma and 1 mm lateral to the midline. A Hamilton syringe with a 27 G needle was used to administer 4 μCi [^3^H]-inositol in 1 μl of inositol (20 mg ml^−1^ in aCSF at a rate of 0.5 μl per 20 s.

### Brain phosphoinositols accumulation

Brain phosphoinositols accumulation was assayed according to Whitworth and Kendall^52^ with minor modifications. In brief, mice were given an ICV injection of 4 μCi [^3^H]-inositol 24 h before tissue extraction. Mice were killed by cervical dislocation followed by immediate decapitation and their brains quickly dissected on ice to separate the frontal cortex. Samples were then sonicated in 1 ml ice-cold perchloric acid (10% w/v) for 20–30 s to extract the [^3^H]-inositol phosphates. Sonicated samples were neutralized with KOH (1.5 m) and left on ice for at least 20 min before centrifugation at 2000 *g* for 20 min. Then, the supernatant was added to 3 ml Tris buffer (50 mm, pH 7.4), mixed and taken for the analysis of total [^3^H]-inositol phosphates accumulation by anion-exchange chromatography on Dowex chloride columns. The columns were washed with 15 ml H_2_O before elution of the [^3^H]-inositol phosphates with 5 ml HCl (1 m). Samples were placed in scintillation vials.

### Incorporation of ^3^H-inositol into brain phosphoinositides

The membranous pellet remaining from the initial extraction (above), after discarding the excess supernatant, was mixed with 0.94 ml chloroform:methanol:6 n HCl (100:200:1) followed by further aliquots of chloroform (0.32 ml) and water (0.32 ml) to extract the [^3^H]-inositol phospholipids. Samples of the chloroform phase containing the phospholipids were transferred into scintillation vials and left to evaporate overnight.

### Obtaining final results of phosphoinositols accumulation and inositol incorporation into brain phosphoinositides

Radioactivity in [^3^H]-inositol phosphates and phospholipids was assessed by liquid scintillation counting. Results were calculated per mg protein in the fraction. Protein concentration was assayed by the Bradford method.^53^

Values obtained following acute and chronic Li treatment were corrected for the well-established reduction in brain inositol levels, ~30% and ~15%, respectively. Similarly, in SMIT1 KO mice, a correction for 60% reduction in inositol levels^39^ was carried out. Values were not corrected for IMPA1 KO mice, as no difference has previously been found in their frontal cortex and hippocampal inositol levels.^28^

### Behavioral tests

The FST and the amphetamine-induced hyperlocomotion test were performed on different cohorts of mice as described elsewhere.^39^

### Administration of IP_3_/IP_1_/IP_3_R_ant_

Each of IP_3_, IP_1_ and IP_3_R_ant_ were administered ICV trapped in liposomes. Liposomes were used to enhance penetration into cells and to protect from rapid dephosphorylation of the inositol phosphates before their transport into the cells. Dose–response experiments of IP_3_ indicated the appropriate dose to be administered. The results of dose–response analyses in the FST appear in the Supplementary Information.

### mRNA and protein levels of autophagy markers

Frontal cortex and hippocampal samples for mRNA and protein extraction were dissected on ice as described before;^47^ mRNA was extracted from mice killed 45 min after IP_3_ or aCSF administration and protein was extracted 45 min or 24 h following IP_3_ or aCSF administration. A pool of all RNA samples was used for normalization. Table 1 summarizes the primer sequences for the genes examined and the respective efficiencies of their reactions.

Protein concentration was determined spectrophotometrically (Nanodrop, Thermo Scientific, Waltham, MA, USA). Western blotting was performed according to our standard protocol for the same proteins.^47^

Autophagy studies frequently use the conversion of microtubule-associated protein1 light chain 3 (LC3)-I to LC3-II as a marker of changes in the process. However, in mouse brain homogenates, only LC3-I is discernible.^54, 55^ We therefore have previously used the ratio between protein levels of two other autophagy markers, Beclin-1 and p62, as readout of autophagy intensity.^47^ The primary antibodies Beclin-1 (#3738, 1:1000, Cell Signaling Technology, Danvers, MA, USA) and p62 (ab56416, 1:1500, Abcam, Cambridge, UK), were diluted in 1 % non-fat dry milk, 1 % bovine serum albumin and 0.01 % sodium azide in Tris-buffered saline Tween 20. Goat anti-rabbit antibodies (sc-2004, 1:10 000, Santa Cruz, Dallas, TX, USA) were diluted in Tris-buffered saline Tween 20. Beclin-1 (encoded by the BECN1) is required for the initiation of the autophagosome formation^56^ and thus is elevated when autophagy is enhanced. p62 (also known as SQSTM1, a ubiquitin-binding scaffold protein^57^) is degraded during the autophagy process and hence its levels are decreased when autophagy is induced.^57^ Thus, an increased Beclin-1/p62 ratio derived from a given sample is suggestive of augmented autophagy.

### Statistical analysis

Statistical analyses of the neurochemical results were carried out using one-way analysis of variance (ANOVA) followed by *post hoc* Fisher's least significant difference (LSD) tests. Statistical analyses of the behavioral experiments were performed using either one-way or two-way ANOVA followed by *post hoc* Fisher's LSD tests as indicated in the Results section. The variance in each type of experiment was found to be similar between the groups that were statistically compared. As given in the figures, all groups in all experiments included 5–38 mice per group. It is our long-lasting experience and common knowledge from the literature that to ensure adequate power to detect a biologically meaningful effect size for neurochemical and behavioral experiments at least five animals per group are required. No animals/samples were excluded from the analyses. All analyses were performed using the STATISTICA XI software (Dell Statistica, Tulsa, OK, USA). Level of statistical significance was set at *P*⩽0.05.

## Results

### ^3^H-phosphoinositols and ^3^H-phosphoinositides 24 h following ICV ^3^H-inositol administration

To better understand whether inositol depletion *per se* or phosphoinositols accumulation mediate Li-induced phenotypes, ^3^H-inositol was administrated ICV to WT-untreated mice, WT mice treated with acute or chronic Li, and to IMPA1 and SMIT1 KO mice. Twenty-four hours later, the levels of ^3^H-phosphoinositols and ^3^H-phosphoinositides were measured in the frontal cortex and hippocampus. Details regarding precaution measurements to assure the measurement of a specific biological effect appear under Supplementary Information/Results. The comparison between WT-untreated mice, WT Li-treated and KO mice was performed separately in each of the two colonies (IMPA1 and SMIT1).

### Phosphoinositols labeling

**Figure 1:**
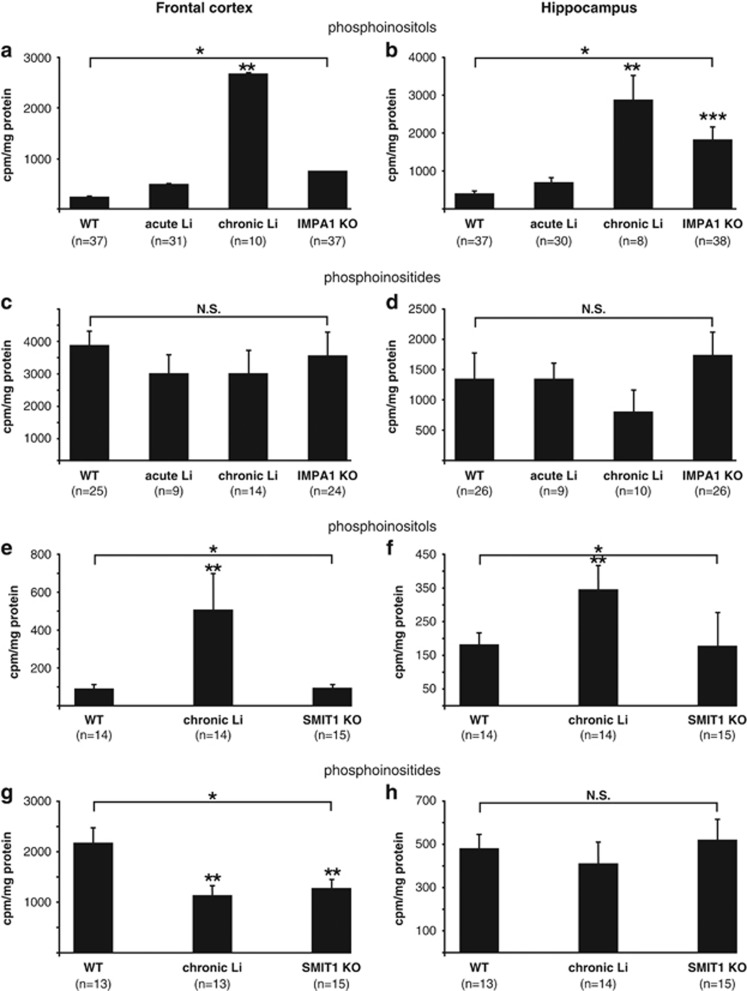
Effect of Li treatment and of KO of IMPA1 and SMIT1 on ^3^H-inositol incorporation into phosphoinositols (**a**, **b**, **e**, **f**) and phosphoinositides (**c**, **d**, **g**, **h**) in the frontal cortex and hippocampus. ^3^H-inositol was administered ICV 24 h before death to WT mice, acutely Li-treated WT mice, chronically Li-treated WT mice, all from the IMPA1 colony, and to IMPA1 KO mice (**a**–**d**) or to WT mice, and chronically Li-treated WT mice, all from the SMIT1 colony, and to SMIT1 KO mice (**e**–**h**), and ^3^H-phosphoinositols accumulation and incorporation of the radiolabeled inositol into ^3^H-phosphoinositides assayed as described under Materials and methods. (**a**) *ANOVA: F(3,111)=38.285, *P*<0.01; Fisher's LSD *post hoc* analysis: acute Li vs WT, *P*=0.1; **chronic Li vs WT, *P*<0.01; ***IMPA1 KO vs WT, *P*=0.002. (**b**) *ANOVA: F(3,109)=13.056, *P*<0.01; Fisher's LSD *post hoc* analysis: acute Li vs WT, *P*=0.37; **chronic Li vs WT, *P*<0.01; ***IMPA1 KO vs WT, *P*<0.01. (**c**) ANOVA: F(3,73)=0.38312, *P*=0.765. (**d**) ANOVA: F(3,67)=0.647, *P*=0.58. (**e**) *ANOVA: F(2,41)=5.8485, *P*<0.01; Fisher's LSD *post hoc* analysis: **chronic Li vs WT, *P*<0.01; SMIT1 KO vs WT, *P*=0.98. (**f**) *ANOVA: F(2,39)=5.0149, *P*=0.01; Fisher's LSD *post hoc* analysis: **chronic Li vs WT, *P*=0.02; SMIT1 KO vs WT, *P*=0.58. (**g**) *ANOVA: F(2,37)=4.932, *P*=0.01; Fisher's LSD *post hoc* analysis: **chronic Li vs WT, *P*<0.01; **SMIT1 KO vs WT, *P*=0.01. (**h**) ANOVA: F(2,40)=0.38, *P*=0.68. ANOVA, analysis of variance; ICV, intracerebroventricularly; KO, knockout; Li, lithium; SMIT1, sodium-*myo*-inositol transporter 1; WT, wild type.

In the IMPA1 colony, both chronic Li treatment of WT mice and KO of IMPA1 resulted in a significantly increased ^3^H-phosphoinositols accumulation. Acute Li treatment induced a non-significant increase and, hence, was not studied in the SMIT1 colony (Figures 1a and b). In the SMIT1 colony, chronic Li treatment but not SMIT1 KO increased phosphoinositols labeling both in the frontal cortex and hippocampus. (Figures 1e and f).

### Phosphoinositides labeling

A different pattern was observed for ^3^H-phosphoinositides accumulation. In the IMPA1 colony, neither Li treatment nor IMPA1 KO affected phosphoinositides labeling (Figures 1c and d). In contrast, in the SMIT1 colony, both chronic Li treatment and SMIT1 KO significantly reduced phosphoinositides labeling in the frontal cortex (Figure 1g). As the IMPA1 and SMIT1 colonies were created on different genetic backgrounds^22, 28^ and the effect of lithium on ^3^H-phosphoinositides accumulation was observed only in mice of the SMIT1 colony, it is possible that this effect is genetic background-dependent. No change was observed in the hippocampus (Figure 1h).

### The effect of IP_3_ on behavior in the FST and amphetamine-induced hyperlocomotion test

**Figure 2:**
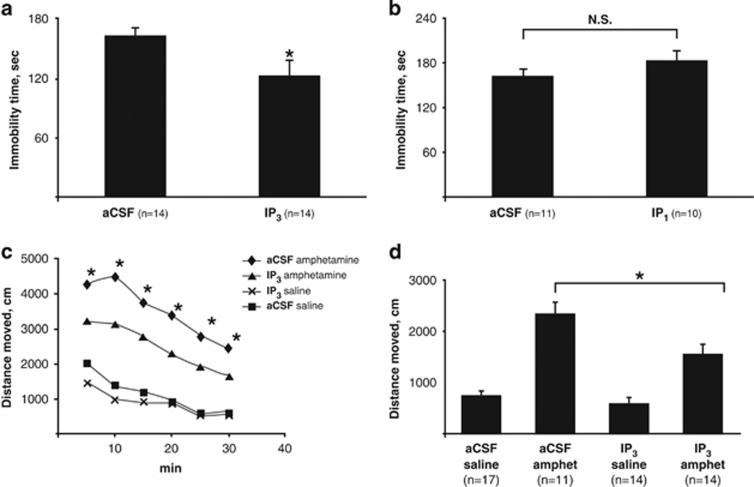
IP_3_ (**a**) but not IP_1_ (**b**) administered ICV to mice reduced immobility time in the FST and IP_3_ attenuated amphetamine-induced hyperlocomotion (**c**, **d**). (**a**, **b**) Immobility time in the FST 45 min following ICV administration of 150 μg IP_3_ (**a**) or 200 μg of IP_1_ (**b**) (or vehicle (aCSF)), each in liposomes. (**a**) *t-test: *t*(26)=5.65, *P*<0.03. (**b**) *t*-test: *t*(19)=1.59, *P*=0.22. (**c**, **d**) Amphetamine-induced hyperlocomotion. Thirty minutes following ICV administration of 150 μg IP_3_ in liposomes, mice were injected (i.p.) 1.5 mg kg^−1^ amphetamine (amphet) or saline, placed in an open-field box and their activity monitored for 30 min. (**c**) Two-way ANOVA: IP_3_ treatment: F(1,52)=55, *P*=0.01; amphetamine treatment: F(1,52)=8.67, *P*<0.01; ***amphetamine × IP_3_ interaction: F(1,51)=4.1, *P*<0.05. (**d**) Two-way ANOVA with repeated measures: IP_3_ treatment: F(6,46)=2.4, *P*=0.03; amphetamine treatment: F(6,46)=13.7, *P*<0.01. Two-way ANOVA for each time interval: *amphetamine/IP_3_ vs amphetamine/aCSF, *P*< at least 0.016. aCSF, artificial cerebrospinal fluid; ANOVA, analysis of variance; FST, forced swim test; IP, inositol phosphate; i.p., intraperitoneal; ICV, intracerebroventricularly; WT, wild type.

We wished to find out whether among the phosphoinositols that accumulate following Li treatment IP_3_, a second messengers in the PI signaling system,^3^ or its metabolite IP_1_, which accumulates due to Li's inhibition of IMPase-1,^7, 8^ mediate Li-induced behavioral changes in two behavioral paradigms: the FST and the amphetamine-induced hyperlocomotion test. Screening experiments of the possible effect of IP_3_ and IP_1_ on motor capabilities are described under Supplementary Information/Results. Similarly to chronic Li treatment,^39, 51, 58^ ICV administration of IP_3_ (trapped in liposomes) resulted, 45 min later, in decreased immobility time in the FST (Figure 2a), an antidepressant-like effect,^59^ and in an attenuated response to amphetamine, an antimanic-like effect^60^ (Figures 2c and d). To further assess whether IP_3_ directly propagates the signal following Li treatment and/or whether the behavioral effects are mediated by its breakdown product, IP_1_, we studied the effect of IP_1_ in a similar manner. An amount of 200 μg of IP_1_ (higher than the effective dose of IP_3_) did not affect the immobility time in the FST as compared with the mice receiving aCSF (Figure 2b). Higher amounts of IP_1_ could not be tested due to toxicity. The possibility that IP_1_ failed to affect the FST due to its effect on motor activity/coordination was ruled out (Supplementary Information/Results). As IP_1_ failed to mimic Li in the FST, we did not study its effect in the amphetamine-induced hyperlocomotion paradigm.

**Figure 3:**
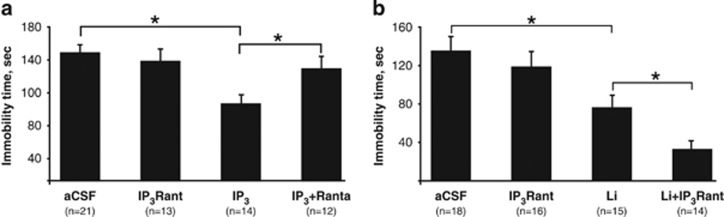
IP_3_R_ant_ administered ICV reversed IP_3_'s effect (**a**) and enhanced Li's effect in the FST (**b**). (**a**, **b**) Immobility time in the FST 45 min following ICV administration of 10 pmol of IP_3_R_ant_ in liposomes. (**a**) Two-way ANOVA: *IP_3_ treatment: F(1,62)=7.98, *P*<0.01; IP_3_R_ant_ treatment: F(1,62)=1.78, *P*=0.18; *IP_3_ treatment × IP_3_R_ant_ treatment interaction: F(1,62)=4.11, *P*=0.046; Fisher's LSD *post hoc* analysis: *IP_3_ vs aCSF, *P*<0.01; *IP_3_+IP_3_R_ant_ vs IP_3_, *P*=0.02. (**b**) Two-way ANOVA: *Li treatment: F(1,59)=27.03, *P*<0.01; *IP_3_R_ant_ treatment: F(1,59)=4.7, *P*=0.03; Li treatment × IP_3_R_ant_ treatment interaction: F(1,59)=0.83, *P*=0.3; Fisher's LSD *post hoc* analysis: *Li+aCSF vs regular food+aCSF, *P*<0.01; *Li+IP_3_R_ant_ vs Li+aCSF, *P*=0.04. aCSF, artificial cerebrospinal fluid; ANOVA, analysis of variance; FST, forced swim test; ICV, intracerebroventricularly; IP, inositol phosphate; IP_3_R_ant_, IP_3_ receptor antagonist.

Given the antidepressant-like and antimanic-like behavioral effects of IP_3_, mimicking well-established effects of Li, a straight-forward assumption was that IP_3_ exerts its behavioral effects through its receptors (IP_3_Rs). Thus, we examined whether the administration of xestospongin-C, an antagonist of all three IP_3_ receptors (IP_3_R_ant_), reverses these effects. Mice were administered ICV with aCSF, or IP_3_, or IP_3_R_ant_ or IP_3_+IP_3_R_ant_, each trapped in liposomes, 45 min before their exposure to the FST. Administration of IP_3_R_ant_ reversed the antidepressant-like effect of IP_3_ (Figure 3a). We further hypothesized that Li's behavioral effect in the FST is also reversed by the IP_3_R_ant_. In contrast with our simplistic hypothesis, Li and xestospongin-C exerted a synergistic effect on the immobility time (Figure 3b).

### The effect of IP_3_ on autophagy markers

IP_3_Rs are involved in the regulation of autophagy,^49^ a cellular process previously shown to be enhanced by Li in an inositol-dependent manner.^15^ As the enhancement of autophagy was shown to induce an antidepressant-like effect,^46, 47^ we tested whether IP_3_ administration also mimics Li treatment at the level of the autophagy process. Autophagy studies frequently use the conversion of microtubule-associated protein1 LC3-I to LC3-II as a marker of changes in the process. However, in mouse brain homogenates, only LC3-I is discernible.^53, 54^ We therefore have previously used the ratio between protein levels of two other autophagy markers, Beclin-1 and p62, as a readout of autophagy intensity.^47^ Beclin-1 (encoded by the *BECN1*) is required for the initiation of the autophagosome formation,^55^ and thus is elevated when autophagy is enhanced. p62 (also known as SQSTM1, a ubiquitin-binding scaffold protein^56^), is degraded during the autophagy process and hence its levels are decreased when autophagy is induced.^56^ Thus, an increased Beclin-1/p62 ratio derived from a given sample is suggestive of augmented autophagy.

**Figure 4:**
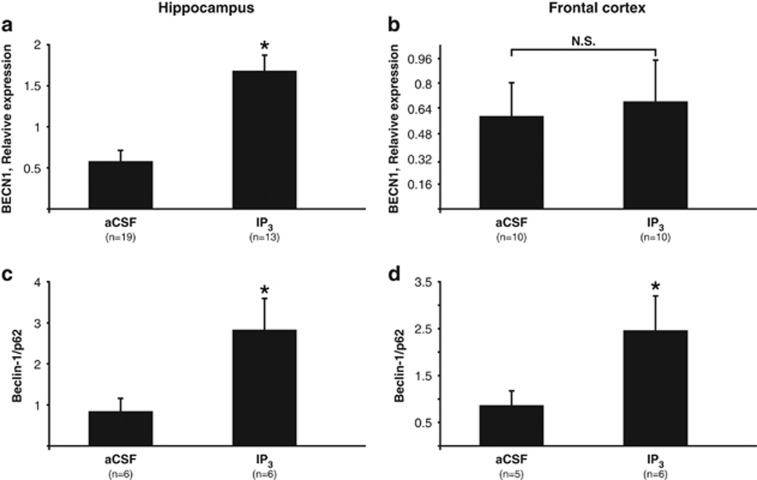
ICV administration of IP_3_ affected autophagy markers suggestive of an enhanced process. 45 min following ICV administration of IP_3_ *BECN1* mRNA levels were elevated in the hippocampus (**a**) but not in frontal cortex (**b**). Twenty-four hours following ICV administration of IP_3_ Beclin-1/p62 protein level ratio was elevated in the hippocampus (**c**) and in the frontal cortex (**d**). (**a**) *t-test, *t*(30)=24.5, *P*<0.01. (**b**) *t*-test, *t*(18)=0.89, *P*=0.35. (**c**) *t-test, *t*(10)=5.95, *P*=0.03. (**d**) *One-tailed *t*-test, *t*(9)=3.21, *P*=0.05. aCSF, artificial cerebrospinal fluid; ICV, intracerebroventricularly; IP, inositol phosphate.

Forty-five minutes following IP_3_ administration, *BECN1* expression was significantly upregulated in the hippocampus but not in the frontal cortex (Figures 4a and b). As protein but not transcript levels of p62 are affected by autophagy, p62 transcript levels were not assessed. Forty-five minutes following IP_3_ administration, Beclin-1/p62 ratio was not affected either in the frontal cortex or in the hippocampus (data not shown). However, 24 h following IP_3_ treatment, the ratio was elevated both in the hippocampus and in the frontal cortex (Figures 4c and d), in a similar manner to chronically Li-treated and of *IMPA1* KO mice: WT, *n*=5, 0.56±0.12 (s.e.m.); Li, *n*=4, 1.67±0.42; *IMPA1* KO, *n*=7, 1.08±0.15; ANOVA, F(2,13)=5.3, *P*<0.025; Fisher's LSD *post hoc* analysis, WT vs Li, *P*<0.03; WT vs *IMPA1* KO, *P*<0.03).

## Discussion

Li perturbs brain inositol metabolism at several sites.^2, 9, 61^ The brain may be particularly Li sensitive due to low inositol penetrability of the blood–brain barrier.^62^ Therefore, the brain relies mainly on the recycling and *de novo* synthesis of inositol.^3^ The response of the PI cycle to Li treatment was extensively investigated using a variety of agonists. However, the reports regarding phosphoinositides and phosphoinositols levels, in general, and IP_3_, in particular, are inconsistent. Namely, *ex vivo* studies in brain slices from Li-treated animals following incubation with [^3^H]-inositol with or without stimulation by receptor agonists and with or without inositol supplementation in the medium reported an increase, a decrease or a lack of effect on [^3^H]-phosphoinositols production.^6, 63, 64^ Elevated IP_3_ levels have been shown in the cerebral cortex of guinea pigs, rabbits, monkeys, rats and mice.^24^ In COS-7 and SK-N-SH cell lines, Sarkar *et al.*^15^ reported the reduction of IP_3_ levels following Li treatment, whereas in the SH-SY5Y cell line, Los *et al.*^65^ found elevated levels in a dose-dependent manner. These inconsistencies might stem from the following reasons: (i) species and cell type differences, for example, the rodent brain exhibits 50% less inositol compared with primate brain;^24, 66^ (ii) while preparing cerebral slices there is 80% loss of inositol and supplementation of 10 mm is required to restore inositol levels.^24^ This might have shifted the equilibrium among the various phosphoinositols. Thus, results obtained from such experiments might not represent the *in vivo* response.

Beyond the different experimental conditions, it could be that the inconsistencies stem from a more complex mechanism of action of Li than reducing the levels of inositol and phosphoinositides, *per se*. We therefore considered that studying basal state PI hydrolysis (without agonist stimulation) might better reflect the response to Li. In accordance with the previous reports,^37, 38^ we found that chronic Li treatment resulted in a significant increase in ^3^H-phosphoinositols accumulation both in the frontal cortex and hippocampus, and acute treatment showed a similar trend. In our previous studies, both IMPA1 and SMIT1 KO mice were shown to exhibit Li-like behavior, but only SMIT1 KOs had reduced brain inositol levels.^28, 29, 39^ This provided the opportunity to decipher whether IP_3_ accumulation and/or inositol depletion are the molecular downstream effects of Li mediating its induced behavior. Interestingly, only IMPA1 KO mice mimicked phosphoinositols accumulation seen following Li treatment. These results suggest that both inositol depletion (according to the results in SMIT1 KOs) and phosphoinositols accumulation (according to the results in IMPA1 KOs) may mediate Li-induced behavioral effects. It is notable that in the frontal cortex of SMIT1 KO mice and chronically Li-treated WT mice from the SMIT1 colony phosphoinositides labeling was significantly decreased. This raises the possibility that the attenuation of inositol turnover also mediates Li-induced behavioral effects.

Taken together, the results support the notion that perturbation of several sites along the PI cycle might mediate behavioral consequences of Li treatment. This concept is different from the original inositol depletion hypothesis that assumed, based on the cyclic nature of the PI signaling system, that inositol metabolites of the cycle are mutually regulated and that by targeting a given site of the cycle all its components are influenced. The current findings suggest that the components previously believed to be metabolically interconnected (phosphoinositols, phosphoinositides and free inositol) have separate modes of action by which they affect behavior, and that perturbing a given component would not necessarily affect the others. This possibility is further supported by clinical studies that reported beneficial therapeutic effects of oral inositol supplementation in illnesses responsive to serotonin-selective re-uptake inhibitors, including depression, panic and obsessive-compulsive disorder,^67^ and as an add-on to the ongoing treatment in BPD.^68^ Either inositol supplementation or inhibition of inositol monophosphatase leading to inositol depletion can, concomitantly, result in IP_3_ accumulation.

The above conclusion should be taken cautiously as our results are based on a crude method of measuring ^3^H-inositol incorporation into soluble and insoluble fractions that were not further separated into specific inositol-containing molecules. In the case of the phosphoinositides, there are different types that carry out different functions.^69^ Similarly, there are several molecular moieties of soluble phosphoinositols, and a number of metabolic pathways that interconvert among themselves (as discussed below).

Accumulation of phosphoinositols following Li treatment and in IMPA1 KO mice raised the question do IP_1_ or IP_3_ mediate the behavioral effects of Li. IP_3_ induced an antidepressant-like and Li-like effect in the FST, and an antimanic-like and Li-like effect in the amphetamine-induced hyperlocomotion paradigm. Non-specific behavioral effects of IP_3_ were ruled out as detailed under Supplementary Information/Results. It is known that IP_1_ accumulation following Li treatment exceeds that of IP_3_.^70^ Nevertheless, IP_1_ did not exhibit an antidepressant-like effect in the FST at a dose similar to that of IP_3_. A threefold higher IP_1_ dose was toxic. It is thus conceivable that IP_3_ is a downstream mediator of Li- and IMPA1 KO- but not SMIT1 KO-induced behavioral effects.

As IP_3_ is known to mediate its effect via binding to IP_3_Rs, we hypothesized that IP_3_-induced behavioral effects are mediated by its receptors that are found on the endoplasmic reticulum. To test this possibility we used the IP_3_R_ant_, xestospongin-C, which antagonizes all three IP_3_Rs.^50^ Similarly to *in vivo* knockdown of IP_3_Rs, high xestospongin-C doses were shown to decrease the immobility time in the FST.^50^ To reduce this confounding effect we chose a xestospongin-C dose that does not influence the behavior of mice in this test. As hypothesized, xestospongin-C reversed IP_3_'s action in the FST, suggesting that Li's behavioral effects mimicked by IP_3_ are mediated via the IP_3_Rs. However, whether all three receptors are involved in these behaviors, one of them or a combination of two, remains to be investigated.

Phosphoinositols with more than three phosphate groups (IP_4_–IP_6_) as well as inositol pyrophosphates (IP_7_ and IP_8_) are also known to exhibit second messenger characteristics. The enzyme phosphatidylinositol-3-kinase (PI_3_K) that produces phosphatidylinositol-3,4,5-trisphosphate (PIP_3_) is also the main enzyme responsible for the generation of inositol pyrophosphates.^71^ We cannot rule out the possibility that IP_3_ is further converted into any of IP_4_–IP_8_ that mediate the behavioral effects induced by administered IP_3_ or by enhanced accumulation following Li treatment. However, this possibility is less likely, as inositol polyphosphates and pyrophosphates are not IP_3_R agonists.

Surprisingly, when xestospongin-C and Li were co-administered, rather than the expected reversal of Li's antidepressant-like effect in the FST, a synergistic effect was obtained. The synergism may be interpreted as follows. Chronic Li was shown to downregulate IP_3_Rs,^72^ possibly as a result of receptor desensitization and internalization following IP_3_ accumulation. The dose of xetospongin-C chosen in the present study (10 pmoles) was, by itself, ineffective in the FST, but higher doses (30 pmoles) were shown to decrease the immobility time in the FST.^50^ Thus, combining the lower xestospongin-C dose with Li induces an effect similar to that of a high xestospongin-C dose, namely, an anti-depressant-like effect. IP_3_R_ant_'s effects could not be reversed by inositol supplementation,^49^ suggesting an inositol-independent pathway, enabling synergism with Li's effect that is supposedly mediated by an inositol-dependent mechanism.

Li has been shown to induce autophagy in an mTOR-independent manner accompanied by reduced IP_3_ levels, both reversed by inositol supplementation.^15^ Rapamycin and trehalose, autophagy inducers, have recently been shown to induce an antidepressant-like decreased immobility in the FST.^46, 47^ Our results indicate that similarly to Li-treated WT mice and IMPA1 KO mice (data submitted for publication), IP_3_ administration upregulated hippocampal but not frontal cortex *BECN1* mRNA levels 45 min after the administration and elevated Beclin-1/p62 protein level ratio both in the hippocampus and frontal cortex 24 h following the administration. Simplistically, these results contradict those of Sarkar *et al.*,^15^ who showed that decreased, rather than elevated, IP_3_ levels promote autophagy. However, it is conceivable that the high dose of IP_3_ that we administered resulted in desensitization of the IP_3_Rs, which have previously been shown to inhibit autophagy by binding Beclin-1.^49^ Thus, similarly to decreased IP_3_ levels, IP_3_ administered ICV led to changes in autophagy markers indicative of enhanced autophagy. Multiple reviews (for example, Morris *et al.*,^73^ Scheuing *et al.*^74^ and Motoi *et al.*^75^) hypothesize the beneficial effects of Li treatment in neurodegenerative disorders based on the reports in animal models and cells in culture of Li-induced enhanced autophagy as a mechanism of the drug's neuroprotective characteristic (for example, Fornai *et al.*,^76^ Fabrizi *et al.*^77^ and Sarkar *et al.*^78^). However, to the best of our knowledge, there are no clinical studies where Li's effect on autophagy was assessed in relation to clinical efficacy.

The fact that the Beclin-1/p62 protein level ratio was elevated 24 h but not 45 min following IP_3_ administration suggests that IP_3_-induced Li-like changes in the levels of autophagy-related proteins occur in parallel rather than upstream to IP_3_-induced Li-like behaviors. However, as demonstrated by Vicencio *et al.*,^49^ in addition to the role of IP3_3_Rs as ion channels, they also function as scaffold proteins for the interaction between Beclin-1 and Bcl2, an interaction that inhibits Beclin-1-induced autophagy. If IP_3_ administration indeed leads to desensitization and internalization of IP_3_Rs, reduced availability of IP_3_Rs would result in decreased interaction between Beclin-1 and Bcl2, and in enhanced autophagy without affecting Beclin-1 levels. Hence, we cannot unequivocally rule out the possibility that 45 min following IP_3_ administration autophagy was increased but not reflected in an increased beclin1/p62 ratio.

**Figure 5:**
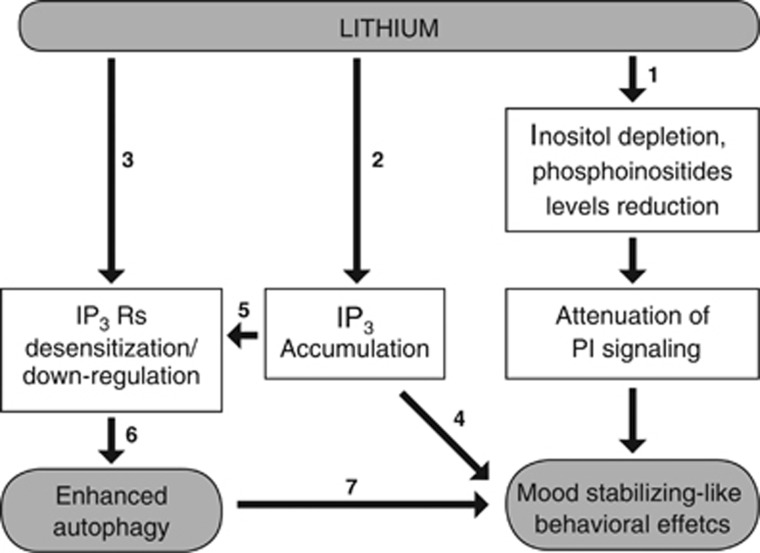
Suggested mechanisms of Li-induced behavior and enhanced autophagy: 1—according to the results in the SMIT1 KO mice; 2—according to the results in the IMPA1 KO mice; 3—according to de Bartolomeis *et al.*;^72^ 4—according to the results in IP_3_-treated mice; 5—a reasonable assumption; 6—according to Vicencio *et al.*;^49^ and 7—according to Kara *et al.*^47^ IP, inositol phosphate; KO, knockout; Li, lithium; PI, phosphatidylinositol.

Taken together, accumulated results in Li-treated mice and transgenic mice (Table 2 and its detailed description), and in IP_3_-treated mice, prompt us to suggest the cascade of events mediating Li's neurochemical, cellular and behavioral effects depicted in Figure 5. Namely, Li-induced behavior may be mediated by reduced inositol levels, or by reduced phosphoinositides levels (according to the results in the SMIT1 KO mice) or by IP_3_ accumulation (according to the results in the IMPA1 KO mice and IP_3_-treated mice). Partially corroborating Sarkar *et al.*,^15^ Li-induced enhanced autophagy may be mediated by IP_3_ accumulation, possibly causing IP_3_Rs desensitization, and by direct IP_3_Rs downregulation.^72^

To sum-up, the present study utilized genetic tools combined with behavioral models, biochemical assays and cellular processes evaluation in an attempt to revisit the inositol depletion hypothesis of Li's molecular mechanism of action. Both phosphoinositols accumulation and reduced phosphoinositides levels were demonstrated. IP_3_ is a highly probable main phosphoinositol but higher phosphorylated inositol moieties may not be ruled out. Whether Li-induced enhanced autophagy mediates the drug's behavioral effects or is a parallel phenomenon requires further investigation.

## Figures and Tables

**Table 1:** Primer sequences and reactions‘ efficiencies

*Gene*	*Sequences*	*Efficiency*
*Beclin-1*	Fw: GAACTCTGGAGGTCTCGCTC	113.75%
	Rev: TAGACCCCTCCATGCCTCAG	
*MAPK6*	Fw: TATCGATGAGGTGCAGCTT	121.35%
	Rev: GTTCTCGTGGTGATCTGGGT	

Abbreviations: Fw, forward; Rev, reverse.

**Table 2:** A comparison among the phenotypes of Li-treated mice, *SMIT1* KO mice and *IMPA1* KO mice

*Measure*	*Li treatment*	*IMPA1 KO*	*SMIT1 KO*
Brain inositol levels	Reduced	Unchanged	Reduced
Brain IP3 accumulation	Elevated	Elevated	Unchanged
Brain phosphoinositide levels	Decreased	Unchanged	Decreased
Autophagy	Enhanced	Enhanced	?
Pilocarpine-induced seizures	Hypersensitive	Hypersensitive	Hypersensitive
FST	Antidepressant-like behavior	Antidepressant-like behavior	Antidepressant-like behavior
Amphetamine-induced hyperlocomotion	Attenuated	?	attenuated

Abbreviations: FST, forced swim test; KO, knockout; Li, lithium.

SMIT1 KO but not IMPA1 KO mice exhibited reduced brain inositol levels observed following Li treatment;^29^ both Li treatment and IMPA1 KO cause enhanced phosphoinositols accumulation (the present study), enhanced autophagy (the present study, and Sarkar *et al.*^15^), as well as hypersensitization to pilocarpine-induced seizures and an antidepressant-like effect in the FST;^29^ both Li-treated mice and SMIT1 KO mice exhibit decreased phosphoinositides labeling (the present study), and SMIT1 KO mice recapitulate the same Li-like behavior in pilocarpine-induced seizures and in the FST and demonstrate, similarly to Li-treated mice, an antimanic-like effect in the amphetamine-induced hyperlocomotion paradigm (unpublished results and Bersudsky *et al.*^39^).
