# Supplementary material for: IP3 accumulation and/or inositol depletion: two downstream lithium's effects that may mediate its behavioral and cellular changes
Source: Transl Psychiatry. 2016 Dec 6;6(12):e968–. doi: 10.1038/tp.2016.217 (PMC5315558; doi:10.1038/tp.2016.217)
Supplement: Supplementary Information [file tp2016217x1.pdf]

IP_3-Accumulation and/or Inositol-Depletion - Two Downstream Lithium's Effects that May Mediate its Behavioral and Cellular Changes

Yeala Sade[1,2,3,4], Lilach Toker[5], Nirit Z. Kara[1,2,3,6], Haim Einat[6], Stanley Rapoport[7], Dieder Moechars[8], Gerard Berry[9], Yuly Bersudsky[2,3,4] and Galila Agam[1,2,3,4]

Supplementary Information

Results

^{3}H-phosphoinositols accumulation 24 hours following ICV ^{3}H-inositol administration represents a biological measure

An issue of concern was the possible confounding effect of postmortem interval (PMI, the time between sacrifice and the extraction of the brain tissue) on our methodology of measuring phosphoinositols accumulation given the labile nature of phosphorylated molecules in the presence of non-specific phosphatases. To rule out such an effect on our results, dissected brains of WT mice were left at room temperature for 0, 30, and 60 minutes prior to the procedure for assaying phosphoinositols accumulation. There was no difference in phosphoinositols labeling at different time points (Supplementary Fig. S1A). Furthermore, an acute Li dose-response experiment in ICR mice showed that 10 mEq/Kg (IP) led to a significantly higher accumulation of ^{3}H-phosphoinositols than 3 or 0 mEq/Kg (Supplementary Fig. S1B). In addition, *IMPA1* homozygous KO mice accumulated significantly more ^{3}H-phosphoinositols than *IMPA1* heterozygous KO mice and WT mice (Supplementary Fig. S1C). The lack of effect of PMI on phosphoinositols accumulation, the positive Li dose-response and *IMPA1* gene dose-response of phosphoinositols accumulation and the additive accumulation following pilocarpine administration to Li-treated mice support the conclusion that we measured a real biological effect and rule out a PMI effect.

The effect of pilocarpine stimulation on Li- and *IMPA1*-KO-induced phosphoinositols accumulation

Pilocarpine is a muscarinic agonist that activates the PI cycle. A robust acute Li effect is to hypersensitize rodents' response to pilocarpine-induced seizures, an effect that can be reversed by the administration of *myo*-inositol in a stereospecific manner (1, 2). WT mice and *IMPA1* KO mice were injected i.p. with LiCl or saline 24 hours prior to the experiment. Four hours later ^{3}H-inositol was

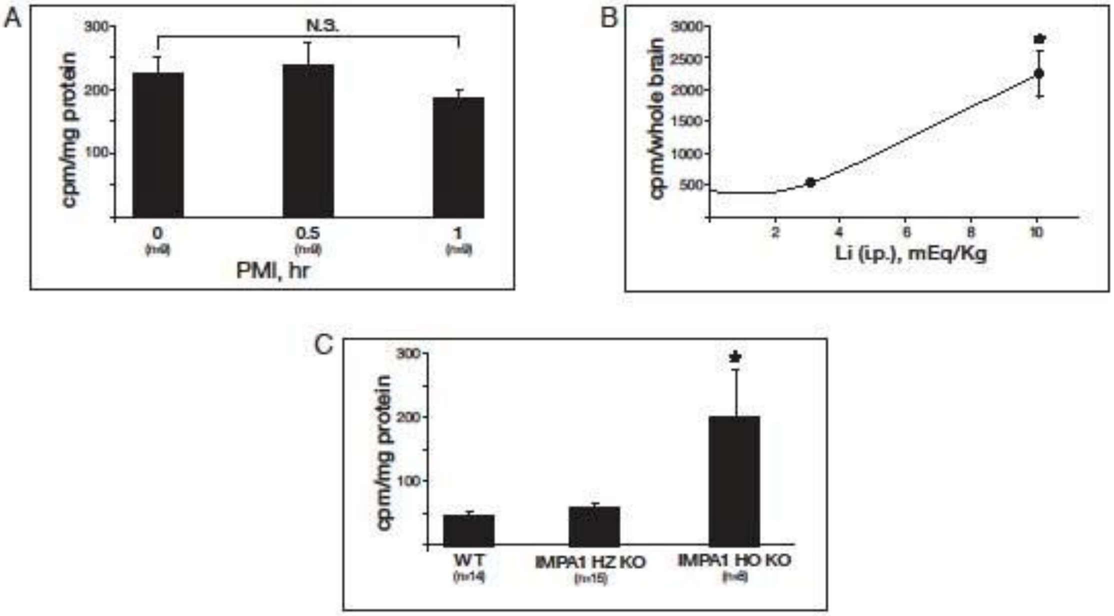

Fig. S1: **The effect of postmortem interval (A), Li dose (B) and extent of *IMPA1* KO (C) on ^{3}H-phosphoinositols accumulation**

A. Dissected brains of WT mice were left at room temperature for 0, 30, and 60 minutes prior to the procedure for assaying phosphoinositols accumulation. PMI upto one hour did not affect phosphoinositols accumulation. ANOVA: $F(2,27)=0.81$, $p=0.45$. **B**. Acutely administered 10 mEq/Kg (IP) Li significantly upregulated phosphoinositols accumulation as compared with 0 and 3 mEq/Kg. *ANOVA: $F(2,34)=20$, $p<0.01$; Fisher's LSD post-hoc analysis: 3 meq/kg *vs.* 0 meq/kg, $p=0.45$; *10 Meq/kg *vs.* 0 meq/kg, $p<0.01$;*10 meq/kg *vs.* 3 meq/kg, $p<0.01$. **C**. The extent of *IMPA1* KO significantly affected phosphoinositols accumulation. *ANOVA: $F(2,37)=5.3$, $p<0.01$; Fisher's LSD post-hoc analysis: heterozygote (HZ) KOs, *vs.* WT $p=0.91$; * homozygote (HO) KOs *vs.* WT, $p<0.01$; *HO *vs.* HZ, $p<0.01$.

injected ICV (as above for 'Acute Li administration'). About half of the animals of each group received a subconvulsive dose of 100 mg/kg pilocarpine (Sigma-Aldrich, St. Louis, MO, dissolved, 10 mg/ml in saline) subcutaneously (s.c.) 15 minutes prior to sacrifice and brain extraction**.** Saline was injected in the control animals. Acute pilocarpine administration increased phosphoinositols labeling in whole brain and in combined cortex and hippocampus (3). We now investigated (a) whether different brain regions, i.e. frontal-cortex and hippocampus, strongly implicated in mood regulation (4), respond in a similar way to pilocarpine-induced phosphoinositols accumulation; (b) whether Li also hypersensitizes this neurochemical effect of pilocarpine; and (c) whether *IMPA1*-KO mice mimic

the effect of Li treatment in their response to pilocarpine. None of the animals showed seizures prior to the time of sacrifice. In the frontal cortex both Li-treated and *IMPA1*-KO mice showed a non-significant ~40% trend towards increased phosphoinositols accumulation (Supplementary Fig. 2A). Pilocarpine further increased phosphoinositols accumulation (280% as compared with WT mice and 210% as compared with Li-treated mice) with no effect on the *IMPA1*-KOs (Supplementary Fig. 2A). In the hippocampus Li treatment and *IMPA1*-KO increased phosphoinositols labeling as compared with WT mice (200% and 70% increase, respectively, Supplementary Fig. 2B) while pilocarpine had no effect (Supplementary Fig. 2B) suggesting brain region differential response to agonist stimulation (5). Alternatively, the different results in the frontal-cortex and hippocampus might stem from a different distribution of muscarinic receptors in these brain regions (6). Despite the similarity between *IMPA1*-KO mice and Li-treated mice in the hypersensitivity to pilocarpine-induced seizures (13) the *IMPA1*-KO mice did not mimic Li treatment regarding phosphoinositols accumulation in response to pilocarpine stimulation. Namely, pilocarpine did not induce further phosphoinositols accumulation neither in the fontal cortex nor in the hippocampus. It is notable that a similar quantitative difference between Litreated mice and *IMPA1*-KO mice in their response to a sub-convulsive dose (100 mg/Kg) of pilocarpine was also observed at the behavioral level. All Li-treated mice seized following pilocarpine administration whereas only 60% of the *IMPA1*-KO mice seized (7).

Dose-response of the effect of IP_3 and IP_3R_{ant} on behavior in the FST

We carried out a dose-response experiment of the effect of ICV administration of IP_3 trapped in liposomes in the FST. IP_3 (Enco, Ann-Arbor, MI, USA, 50, 100 or 150 µg), dissolved in artificial cerebrospinal fluid (aCSF) and trapped in liposomes [Liposomes kit: lipid mixtures for the preparation of liposomes (Sigma-Aldrich, St. Louis, MO)], was administered ICV. Liposomes were prepared according to the manufacturer's instructions with slight modifications as we have previously described (8, 9) to avoid spontaneous seizures induced by the liposome suspensions. Reconstituted liposomes were mixed 1:5 with either aCSF or IP_3 followed by a short sonication for 5 sec using a Polytron cell homogenizer (Heat System Ultrasonic, Newtown, CT, USA) at 50% power capacity. Mice were tested in the FST 45 min following IP_3 administration (Supplementary Fig. S1A). Based on the results of the dose-response experiment we selected the 150 µg IP_3 dose for later experiments.

We also carried out a dose-response experiment for the effect of 5, 10 and 30 pmoles of xestospongin-C in the FST. As previously reported (10), 30 pmoles xestospongin-C administered ICV in liposomes 45 min prior to the test reduced the immobility time (Supplementary Fig. S3B). Based on

the results we selected the 10 pmole dose for later experiments because it was the highest dose that did not have an effect in the FST.

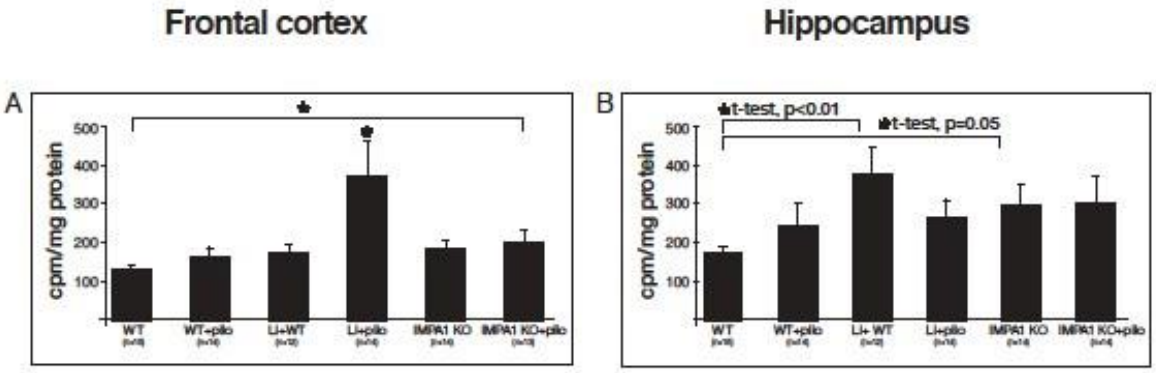

Fig S2. **The effect of the muscarinic agonist pilocarpine on phosphoinositols accumulation in the frontal cortex (A) and in the hippocampus (B)**

A. Pilocarpine augmented the effect of acute Li treatment, but not *IMPA1*-KO on frontal cortex phosphoinositols accumulation. ANOVA: $F(5,76)=4.02$, $p<0.01$; Fisher's LSD post-hoc analysis: WT+pilocarpine *vs*. WT, $p=0.56$; Li *vs*. WT, $p=0.44$; *IMPA1* KO *vs*. WT, $p=0.31$; *Li+pilocarpine *vs*. Li, $p<0.01$; *Li+pilocarpine *vs*. WT+pilocarpine, $p<0.01$; *IMPA1* KO+pilocarpine *vs*. *IMPA1* KO, $p=0.79$.

B. Pilocarpine did not affect the effect of acute Li treatment or *IMPA1*-KO on hippocampal phosphoinositols accumulation. * Li *vs*. WT, t-test: $t(25)=8.43$, $p<0.01$; **IMPA1* KO *vs*. WT, t-test: $t(25)=4.1$, $p=0.05$. pilo=pilocarpine

The effect of 150 μg IP_3 and of 200 μg IP_1 on motor capabilities

To rule out possible confounding effects of the selected IP_3 and IP_1 doses on motor capabilities of ICR mice we analyzed whether 150 μg of IP_3 or 200 μg of IP_1 (a separate cohort of animals) compared with that of aCSF, each trapped in liposomes, when administered ICV 45 min prior to the assessment affect motor activity and coordination (Supplementary Fig. 4). For the open field test mice were placed individually in monitor boxes (37.5x37.5x45cm) and their behavior digitally reordered. Distance moved was analyzed using the Noldus EthoVision system (Wageningen, the Netherlands).

Neither IP_3 nor IP_1 affected motor activity in the open field test (Supplementary Fig. 4A, B). For the beam walking test mice were placed on a beam at the end of which there was food and their home cage. We measured how long it took the mice to get to the food. The test ended when the mice reached

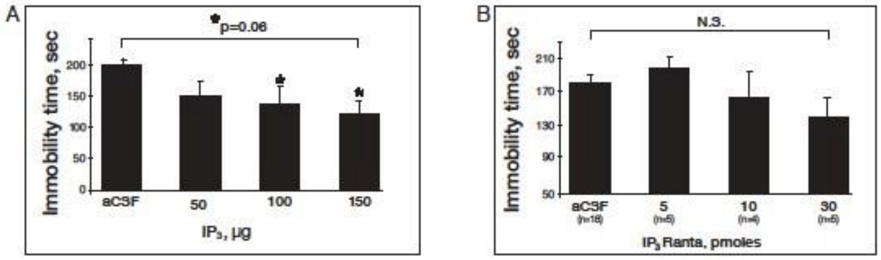

Fig. S3: **Dose response of the effect of IP_3 (A) and IP_3R_{ant} (B) on the behavior in the FST**
IP_3 and IP_3R_{ant} were administered ICV trapped in liposomes. Immobility time in the FST was monitored 45 minutes following the administration. **A**.*ANOVA: $F(3,16)=2.99$, $p=0.06$. Fisher's LSD post-hoc analysis: 50 µg IP_3 *vs.* aCSF, $p=0.1$; *100 µg IP_3 *vs.* aCSF, $p=0.04$; *150 µg IP_3 *vs.* aCSF, $p=0.01$. A dose of 150 µg IP_3 was chosen for further experiments. **B**. ANOVA: $(3,24)=1.9$, $p=0.156$. A dose of 10 pmols of IP_3Rant was chosen for further experiments.

the food or if they fell from the beam. Neither IP_3 nor IP_1 altered the balance of the mice in the beam walking test (Supplementary Fig. 4C, D). For the hanging-wire test each animal was placed on a metal grid that was gently shaken to cause a grabbing reflex, and slowly turned upside down. Latency to fall was counted during a session of 1 min, as previously described (11). IP_3 but not IP_1 decreased their strength in this paradigm (Supplementary Fig. 4E, F). Hence, non-specific behavioral effects of IP_3 were ruled out because of the lack of the compound's effect in the open-field and the beam walking tests. The significant effect of IP_3 in the hanging wire test was disregarded since it was counterintuitive with respect to the FST results in which the mice were more, rather than less, mobile as compared with the control group.

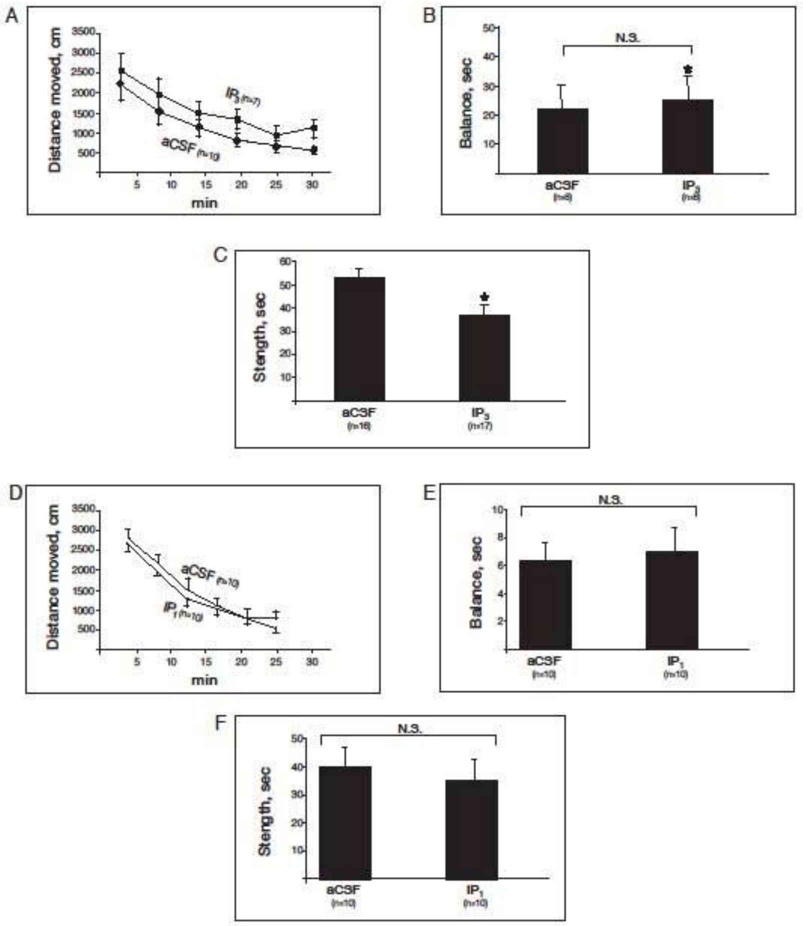

Fig S4: **Effect of ICV administration of IP_3 or of IP_1 in liposomes on motor activity and coordination**

150 µg IP_3 or 200 µg IP_1 or aCSF each trapped in liposomes were administered ICV. **A** & **D**. 30 min after the ICV administration motor activity (distance moved) was measured in an open field box for 30 min. Repeated measures ANOVA: **A**. $F(1,15)=1.63$, $p=0.22$. **D**. $F(1,14)=0.45$, $p=0.51$. **B,C & E,F**. 45 minutes later mice were tested for balance (B&E) and strength (C&F). **B**. t-test: $t(14)=0.06$, $p=0.81$; **C**. *t-test: $t(31)=7.4$, $p=0.01$; **E**. t-test: $t(18)=0.03$, $p=0.84$; **F**. t-test: $t(18)=0.4$, $p=0.53$.

150 µg IP_3 did not affect the behavior of the mice either in the open field or in the balance test. It significantly reduced the strength of the mice. 200 µg IP_1 did not affect any of the tests.

References

1. Belmaker RH, Bersudsky Y. Lithium-pilocarpine seizures as a model for lithium action in mania. Neurosci Biobehav Rev. 2007;31(6):843-9.
2. Kofman O, Belmaker RH. Ziskind-Somerfeld Research Award 1993. Biochemical, behavioral, and clinical studies of the role of inositol in lithium treatment and depression. Biological psychiatry. 1993;34(12):839-52. Epub 1993/12/15.
3. Whitworth P, Heal DJ, Kendall DA. The effects of acute and chronic lithium treatment on pilocarpine-stimulated phosphoinositide hydrolysis in mouse brain in vivo. British journal of pharmacology. 1990;101(1):39-44. Epub 1990/09/01.
4. Chen CH, Suckling J, Lennox BR, Ooi C, Bullmore ET. A quantitative meta-analysis of fMRI studies in bipolar disorder. Bipolar disorders. 2011;13(1):1-15. Epub 2011/02/16.
5. Fisher SK, Bartus RT. Regional differences in the coupling of muscarinic receptors to inositol phospholipid hydrolysis in guinea pig brain. Journal of neurochemistry. 1985;45(4):1085-95.
6. Yamamura HI, Kuhar MJ, Snyder SH. In vivo identification of muscarinic cholinergic receptor binding in rat brain. Brain research. 1974;80(1):170-6.
7. Cryns K, Shamir A, Van Acker N, Levi I, Daneels G, Goris I, et al. IMPA1 is essential for embryonic development and lithium-like pilocarpine sensitivity. Neuropsychopharmacology. 2008;33(3):674-84.
8. Levi I, Eskira Y, Eisenstein M, Gilon C, Hoffman A, Talgan Y, et al. Inhibition of inositol monophosphatase (IMPase) at the calbindin-D28k binding site: molecular and behavioral aspects. European neuropsychopharmacology : the journal of the European College of Neuropsychopharmacology. 2013;23(12):1806-15. Epub 2013/04/27.
9. Shtein L, Toker L, Bersudsky Y, Belmaker RH, Agam G. The inositol monophosphatase inhibitor L-690,330 affects pilocarpine-behavior and the forced swim test. Psychopharmacology. 2013;227(3):503-8. Epub 2013/01/25.
10. Galeotti N, Vivoli E, Norcini M, Bartolini A, Ghelardini C. An antidepressant behaviour in mice carrying a gene-specific InsP3R1, InsP3R2 and InsP3R3 protein knockdown. Neuropharmacology. 2008;55(7):1156-64. Epub 2008/08/19.
11. Crawley JN. Behavioral phenotyping of transgenic and knockout mice: experimental design and evaluation of general health, sensory functions, motor abilities, and specific behavioral tests. Brain research. 1999;835(1):18-26. Epub 1999/08/17.
